# Yield performance of machine-transplanted double-season rice grown following oilseed rape

**DOI:** 10.1038/s41598-019-43348-7

**Published:** 2019-05-02

**Authors:** Min Huang, Alin Tian, Xuefeng Zhou, Wei Gao, Zhibin Li, Ge Chen, Zusheng Li, Yumei Chen, Longsheng Liu, Xiaohong Yin, Yingbin Zou

**Affiliations:** 1 Southern Regional Collaborative Innovation Center for Grain and Oil Crops (CICGO), Hunan Agricultural University, Changsha, 410128 China; 2 Yueyang Academy of Agricultural Sciences, Yueyang, 414000 China; 3 Hengyang Institute of Agricultural Sciences, Hengyang, 421101 China; 4 Guangxi Key Laboratory of Rice Genetics and Breeding, Rice Research Institute, Guangxi Academy of Agricultural Sciences, Nanning, 530007 China

**Keywords:** Agroecology, Sustainability

## Abstract

Growing oilseed rape in the fallow season may be a feasible alternative to growing green manure (e.g. Chinese milk vetch) for improving rice productivity. The objective of this study was to determine the yield performance of machine-transplanted double-season rice (i.e. early- and late-season rice) grown following oilseed rape. Field experiments were conducted to compare machine-transplanted double-season rice grown following oilseed rape, Chinese milk vetch and fallow (i.e. no crop) at Hengyang and Yueyang, Hunan Province, China in three cropping cycles from 2014 to 2017. Results showed that machine-transplanted double-season rice grown following oilseed rape and Chinese milk vetch produced similar grain yield, which was higher than that grown following fallow across two sites and three cropping cycles. The higher grain yield of machine-transplanted double-season rice grown following oilseed rape and Chinese milk vetch was attributable to improvement in both sink size (spikelet number per m^2^) and source capacity (total biomass). However, the reasons for the improved sink size of machine-transplanted double-season rice grown following oilseed rape and Chinese milk vetch were not entirely the same. Growing oilseed rape increased panicle size (spikelet number per panicle) and panicle number in early- and late-season rice, respectively, while growing Chinese milk vetch increased panicle number in both the early- and late-season rice. Our study suggests that growing oilseed rape in the fallow season is a useful alternative strategy for improving productivity of machine-transplanted double-season rice.

## Introduction

Rice is the major staple food crop for more than half of the world’s population, and more than 90% of rice worldwide is produced and consumed in Asia^1^. The intensification of rice cropping systems has helped ensure production of sufficient food in Asia^2^, and a further increase in cropping intensity is considered an important approach for achieving higher food security in the future^3^. However, the continuous rice cropping systems practiced for several decades have led to declines in productivity and raised concerns about sustainability^4,5^. Compared to continuous cropping systems, well-planned crop rotations are expected to promote nutrient cycling efficiency, effective use of natural resources, control of soil-borne pathogens, maintenance of long-term land productivity, and consequently increase crop yields and sustainability of cropping systems^6^.

China is the largest rice consumer in the world, accounting for a third of global rice consumption^7^. In order to produce enough rice, double-season rice (i.e. early- and late-season rice) based cropping systems have been extensively developed in southern China^8^. In recent years, China’s rice production has entered a transition period, during which the government has been gradually promoting large-scale farming^9,10^. The development of large-scale farming has accelerated the adoption of mechanized production techniques for rice in China.

Growing green manure crops (e.g. Chinese milk vetch) in the fallow season is a traditional practice used in rice production in China^11^, and this practice has been clearly documented as beneficial for improving rice productivity^12–14^. However, in recent years, many Chinese farmers have had little enthusiasm to grow green manure crops, because (1) no direct economic return is obtained from growing green manure; and (2) a certain amount of labor is required to grow green manure. In addition, urban expansion has led to a labor shortage and an increase in labor wages in rural areas^11,15,16^. As a consequence, the planting area of green manure has sharply decreased from about 13 million ha in the 1970s to about 2 million ha in the 2010s in China^15^.

Oilseed rape may be a feasible alternative to green manure in Chinese rice cropping systems, since it is not only an excellent rotation crop for cereals but also a cash crop^17^. Our previous studies indicate that oilseed rape can offer several rotational benefits in single-season rice cropping systems, including improving soil fertility, increasing the earthworm population, reducing dependence on external fertilizer inputs and maintaining high rice productivity^18–20^. In recent years, the benefits of growing oilseed rape have been identified in major double-season rice cropping provinces in China such as Hunan, and great progress has been made in developing high-yielding short-duration oilseed rape cultivars for the double-season rice cropping system^21^. However, there is limited information available on how growing oilseed rape in the fallow season affects crop performance of double-season rice.

In the present study, field experiments were conducted to compare machine-transplanted double-season rice grown following oilseed rape, Chinese milk vetch and fallow (i.e. no crop) at two sites in three cropping cycles. The objective of this study was to determine the yield performance of machine-transplanted double-season rice grown following oilseed rape.

## Results

### Grain yield

Grain yield of early-season rice ranged from 7.03 to 8.67 t ha^−1^ with a mean of 7.69 t ha^−1^ under ODR (Table 1). There was no significant difference in mean grain yield of early-season rice between ODR and MDR. Mean grain yield of early-season rice was about 5% higher under ODR and MDR than under FDR. Grain yield of late-season rice under ODR ranged from 6.00 to 8.36 t ha^−1^ with a mean of 7.04 t ha^−1^. The difference in mean grain yield of late-season rice was not significant between ODR and MDR. Mean grain yield of late-season rice was higher under ODR and MDR than under FDR by 5% and 8%, respectively.

**Table 1:** Grain yield (t ha^−1^) of machine-transplanted double-season rice grown under three cropping systems at two sites in three cropping cycles.

Cropping system^†^	Site	Cropping cycle	Early-season rice	Late-season rice
ODR	Hengyang	2014–2015	7.37	5.72
		2015–2016	8.67	8.36
		2016–2017	8.27	6.76
	Yueyang	2014–2015	7.50	7.01
		2015–2016	7.28	8.36
		2016–2017	7.03	6.00
		Mean	7.69a	7.04a
MDR	Hengyang	2014–2015	7.71	6.21
		2015–2016	8.44	8.45
		2016–2017	8.56	7.16
	Yueyang	2014–2015	7.95	7.47
		2015–2016	7.20	8.37
		2016–2017	6.13	5.96
		Mean	7.66a	7.27a
FDR	Hengyang	2014–2015	7.52	5.53
		2015–2016	8.34	8.28
		2016–2017	7.05	6.88
	Yueyang	2014–2015	7.50	7.08
		2015–2016	7.15	7.14
		2016–2017	6.35	5.47
		Mean	7.32b	6.73b

^†^ODR, oilseed rape followed by double-season rice; MDR, Chinese milk vetch followed by double-season rice; FDR, fallow followed by double-season rice.

Within a column, means of cropping systems followed by the same letters are not significantly different according to LSD (0.05).

### Yield components

Panicle number per m^2^ of early-season rice under ODR ranged from 246 to 354 with a mean of 289 (Table 2). Mean panicle number per m^2^ of early-season rice was 7% lower under ODR than under MDR. There was no significant difference in mean panicle number per m^2^ of early-season rice between ODR and FDR. Mean panicle number per m^2^ of early-season rice under MDR was 7% higher than under FDR. Panicle number per m^2^ of late-season rice ranged from 236 to 314 with a mean of 261 under ODR. Mean panicle number per m^2^ of late-season rice under ODR was lower than under MDR and higher than under FDR, but the differences were not significant. Mean panicle number per m^2^ of late-season rice was 11% higher under MDR than under FDR.

**Table 2:** Yield components of machine-transplanted double-season rice grown under three cropping systems at two sites in three cropping cycles.

Cropping system^†^	Site	Cropping cycle	Early-season rice					Late-season rice				
			Panicles m^−2^	Spikelets panicle^−1^	Spikelets m^−2^ (×10^3^)	Spikelet filling (%)	Grain weight (mg)	Panicles m^−2^	Spikelets panicle^−1^	Spikelets m^−2^ (×10^3^)	Spikelet filling (%)	Grain weight (mg)
ODR	Hengyang	2014–2015	273	128	34.9	63.8	28.5	236	140	33.0	76.3	27.6
		2015–2016	354	138	48.9	64.1	27.7	240	165	39.6	69.4	29.0
		2016–2017	276	141	38.9	74.1	28.6	276	148	40.8	59.9	27.8
	Yueyang	2014–2015	302	118	35.6	81.6	28.9	314	123	38.6	65.1	28.7
		2015–2016	283	134	37.9	69.9	26.5	246	161	39.6	77.4	27.4
		2016–2017	246	103	25.3	90.5	29.7	254	127	32.3	72.6	27.2
		Mean	289b	127a	36.9a	74.0a	28.3a	261ab	144a	37.3a	70.1a	28.0a
MDR	Hengyang	2014–2015	292	125	36.5	61.4	28.0	245	140	34.3	79.3	27.5
		2015–2016	384	129	49.5	58.1	27.7	242	167	40.4	65.8	29.0
		2016–2017	306	133	40.7	76.4	28.3	310	142	44.0	62.1	27.9
	Yueyang	2014–2015	321	119	38.2	75.6	28.5	335	130	43.6	64.8	28.5
		2015–2016	292	129	37.7	68.0	25.8	259	155	40.1	76.2	27.7
		2016–2017	277	108	29.9	90.0	29.5	251	126	31.6	72.1	27.7
		Mean	312a	123ab	38.8a	71.6b	28.0b	274a	143a	39.0a	70.0a	28.1a
FDR	Hengyang	2014–2015	285	116	33.1	63.1	28.3	231	117	27.0	78.0	27.6
		2015–2016	400	119	47.6	64.3	27.6	234	156	36.5	71.2	29.1
		2016–2017	263	134	35.2	76.8	29.1	272	132	35.9	63.7	27.8
	Yueyang	2014–2015	272	115	31.3	81.7	29.1	319	120	38.3	64.3	29.0
		2015–2016	274	136	37.3	72.1	27.0	218	183	39.9	74.7	27.6
		2016–2017	259	94	24.3	91.2	29.6	208	125	26.0	74.5	27.2
		Mean	292b	119b	34.8b	74.9a	28.5a	247b	139a	33.9b	71.1 a	28.0a

^†^ODR, oilseed rape followed by double-season rice; MDR, Chinese milk vetch followed by double-season rice; FDR, fallow followed by double-season rice.

Within a column, means of cropping systems followed by the same letters are not significantly different according to LSD (0.05).

Spikelet number per panicle of early-season rice ranged from 103 to 141 with a mean of 127 under ODR (Table 2). Mean spikelet number per panicle of early-season rice under ODR was not significantly higher than under MDR but was significantly (7%) higher than under FDR. Mean spikelet number per panicle of early-season rice under MDR was higher than under FDR, but the difference was not significant. Spikelet number per panicle of late-season rice under ODR ranged from 123 to 165 with a mean of 144. There was no significant difference in mean spikelet number per panicle of late-season rice among ODR, MDR and FDR.

Spikelet number per m^2^ of early-season rice under ODR ranged from 25.3 × 10^3^ to 48.9 × 10^3^ with a mean of 36.9 × 10^3^ (Table 2). There was no significant difference in mean spikelet number per m^2^ of early-season rice between ODR and MDR. Mean spikelet number per m^2^ of early-season rice was 6% and 11% higher under ODR and MDR, respectively, than under FDR. Spikelet number per m^2^ of late-season rice ranged from 32.3 × 10^3^ to 40.8 × 10^3^ with a mean of 37.3 × 10^3^ under ODR. The difference in mean spikelet number per m^2^ of late-season rice was not significant between ODR and MDR. Mean spikelet number per m^2^ of late-season rice was higher under ODR and MDR than under FDR by 10% and 15%, respectively.

Spikelet filling percentage of early-season rice ranged from about 64% to 91% with a mean of 74% under ODR (Table 2). Mean spikelet filling percentage of early-season rice under ODR was slightly (about 2%) but significantly higher than that under FDR. There was no significant difference in mean spikelet filling percentage of early-season rice between ODR and FDR. Mean spikelet filling percentage of early-season rice was slightly (about 3%) but significantly lower under MDR than under FDR. Spikelet filling percentage of late-season rice under ODR ranged from about 60% to 77% with a mean of about 70%. The difference in mean spikelet filling percentage of late-season rice was not significant among ODR, MDR and FDR.

Grain weight of early-season rice under ODR ranged from 26.5 to 29.7 mg with a mean of 28.3 mg (Table 2). Mean grain weight of early-season rice was slightly (1%) but significantly higher under ODR than under FDR. The difference in mean grain weight of early-season rice was not significant between ODR and FDR. Mean grain weight of early-season rice under MDR was slightly (2%) but significantly lower than under FDR. Grain weight of late-season rice ranged from 27.2 to 29.0 mg with a mean of 28.0 mg under ODR. There was no significant difference in mean grain weight of late-season rice among ODR, MDR and FDR.

### Total biomass and harvest index

Total biomass of early-season rice ranged from 976 to 1433 g m^−2^ with a mean of 1215 g m^−2^ under ODR (Table 3). Mean total biomass of early-season rice under ODR (1215 g m^−2^) was lower than that under MDR (1258 g m^−2^) and higher than that under FDR (1162 g m^−2^), but the differences were not significant. Mean total biomass of early-season rice under MDR was 8% higher than that under FDR. Total biomass of late-season rice under ODR ranged from 1105 to 1319 g m^−2^ with a mean of 1226 g m^−2^. The difference in mean total biomass of late-season rice was not significant between ODR (1226 g m^−2^) and MDR (1283 g m^−2^). Mean total biomass of late-season rice was higher under ODR and MDR than under FDR (1139 g m^−2^) by 8% and 13%, respectively.

**Table 3:** Total biomass and harvest index of machine-transplanted double-season rice grown under three cropping systems at two sites in three cropping cycles.

Cropping system^†^	Site	Cropping cycle	Early-season rice		Late-season rice	
			Total biomass (g m^−2^)	Harvest index	Total biomass (g m^−2^)	Harvest index
ODR	Hengyang	2014–2015	1154	0.47	1130	0.52
		2015–2016	1433	0.52	1319	0.51
		2016–2017	1281	0.56	1264	0.46
	Yueyang	2014–2015	1245	0.58	1247	0.50
		2015–2016	1202	0.50	1292	0.56
		2016–2017	976	0.60	1105	0.50
		Mean	1215ab	0.54a	1226a	0.51a
MDR	Hengyang	2014–2015	1176	0.46	1196	0.54
		2015–2016	1470	0.46	1326	0.50
		2016–2017	1377	0.55	1376	0.47
	Yueyang	2014–2015	1266	0.56	1418	0.49
		2015–2016	1141	0.49	1300	0.55
		2016–2017	1119	0.61	1083	0.50
		Mean	1258a	0.52b	1283a	0.51a
FDR	Hengyang	2014–2015	1105	0.46	935	0.53
		2015–2016	1480	0.49	1261	0.51
		2016–2017	1173	0.57	1147	0.48
	Yueyang	2014–2015	1115	0.57	1250	0.49
		2015–2016	1182	0.53	1293	0.55
		2016–2017	916	0.62	951	0.48
		Mean	1162b	0.54a	1139b	0.51a

^†^ODR, oilseed rape followed by double-season rice; MDR, Chinese milk vetch followed by double-season rice; FDR, fallow followed by double-season rice.

Within a column, means of cropping systems followed by the same letters are not significantly different according to LSD (0.05).

Harvest index of early-season rice under ODR ranged from 0.47 to 0.60 with a mean of 0.54 (Table 3). Mean harvest index of early-season rice under ODR was 4% higher than that under MDR. The difference in mean harvest index of early-season rice was not significant between ODR and FDR. Mean harvest index of early-season rice was 4% lower under MDR than under FDR. Harvest index of late-season rice under ODR ranged from 0.46 to 0.56 with a mean of 0.51. There was no significant difference in mean harvest index of late-season rice among ODR, MDR and FDR.

## Discussion

Our study showed that machine-transplanted double-season rice grown following oilseed rape and Chinese milk vetch produced similar grain yields that were higher than rice grown following fallow across two sites and three cropping cycles. This finding is consistent with previous studies showing that (1) oilseed rape is a useful rotation crop for maintaining high productivity of single-season rice^19^; and (2) growing Chinese milk vetch in the fallow season is a feasible practice to sustain rice productivity in both double- and single-season rice cropping systems^12–14^. However, more importantly, our finding suggests that growing oilseed rape can be an alternative strategy to growing Chinese milk vetch in the fallow season for improving productivity of machine-transplanted double-season rice.

The higher grain yield of machine-transplanted double-season rice grown following oilseed rape and Chinese milk vetch was attributable to improvement in both sink size and source capacity. For the sink size, spikelet number per m^2^ was higher in machine-transplanted double-season rice grown following oilseed rape and Chinese milk vetch than in rice grown following fallow. For the source capacity, machine-transplanted double-season rice grown following oilseed rape and Chinese milk vetch produced higher biomass than rice grown following fallow. The importance of simultaneously improving sink size and source capacity in increasing rice yield has been reported in several previous studies^22–24^.

Improvement in sink size can be achieved by increasing panicle number or panicle size (spikelet number per panicle) or both^23^. The results of this study showed that the reasons for the improved sink size of machine-transplanted double-season rice grown following oilseed rape and Chinese milk vetch were not entirely the same. Growing oilseed rape increased panicle size and panicle number in early- and late-season rice, respectively, while growing Chinese milk vetch increased only panicle number in both the early- and late-season rice. This finding indicates that the effects of growing oilseed rape in the fallow season on machine-transplanted double-season rice plants occurred later than effects of growing Chinese milk vetch. This might be related to the fact that decomposition and nutrient release from oilseed rape straw were slower than those from Chinese milk vetch. The results of this study highlight the need for a fundamental understanding of the decomposition and nutrient release processes of oilseed rape straw and their relationships with plant growth of machine-transplanted double-season rice. The results of this study also suggest that further investigations are required to examine the effect of growing oilseed and Chinese milk vetch on yield components in machine-transplanted double-season rice using cultivars with different tillering capacities and panicle sizes.

In addition, results of this study showed that there was a compatible relationship between panicle number and panicle size in machine-transplanted double-season rice grown following oilseed rape and Chinese milk vetch. This finding is inconsistent with previous studies, such as Ying *et al*.^23^ and Huang *et al*.^25^, who reported that there was a compensation effect between the two yield components: if there was more of one, there was less of the other. However, increasing biomass production is a feasible way to decouple the compensations among yield components in cereals including rice^22^. In this study, the higher biomass production could also be responsible for the compatible relationship between panicle number and panicle size in machine-transplanted double-season rice grown following oilseed rape and Chinese milk vetch.

A recent study, conducted by Xie *et al*.^13^, investigated the effects of growing Chinese milk vetch on soil and physiological processes governing plant growth of the subsequent rice crop. Their results showed that growing Chinese milk vetch can improve soil N conservation and recovery, increase leaf chlorophyll content, alleviate oxidative damage to plants, and consequently promote biomass production of the subsequent rice crop. This might also be responsible for the higher biomass production of machine-transplanted double-season rice grown following Chinese milk vetch in this study. However, there is limited information available on the critical soil and physiological factors that explain the higher biomass production in machine-transplanted double-season rice grown following oilseed rape, and therefore further investigations are needed to determine these factors.

Significant decreases in spikelet filling percentage, grain weight and harvest index were observed in the early-season rice grown following Chinese milk vetch as compared to rice grown after oilseed rape and fallow. This finding indicates that a reduction in assimilate partitioning to the grain occurred in the early-season rice grown following Chinese milk vetch. It is well documented that assimilate partitioning is closely related to plant senescence in rice, and early senescence can increase assimilate partitioning to grains^26^. The reduced assimilate partitioning to the grain in the early-season rice grown following Chinese milk vetch might be attributed to delayed plant senescence due to increased N uptake^13^. This could be supported by results reported by Zhou *et al*.^14^, who observed that integration of growing Chinese milk vetch with reducing N fertilizer rate could increase assimilate partitioning to grains in double-season rice.

**Figure 1:**
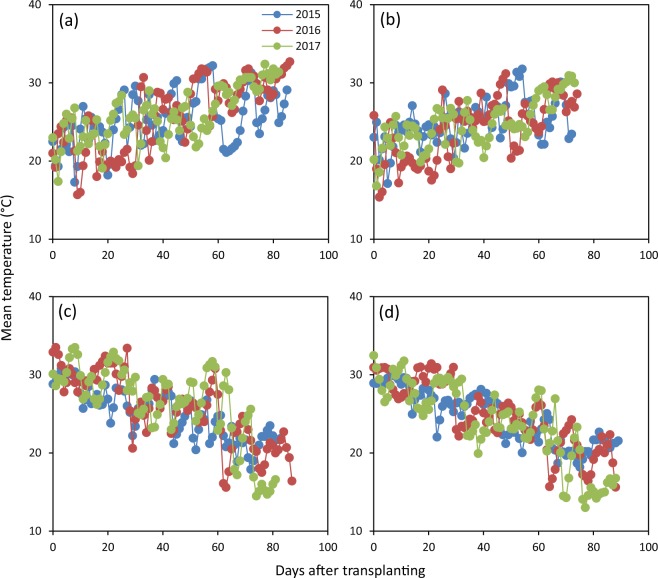
Daily mean temperature during the early (**a**,**b**) and late rice-growing season (**c**,**d**) at Hengyang (**a**,**c**) and Yueyang (**b**,**d**) in 2015–2017. The data were obtained from the local weather bureau at Hengyang and collected by an on-site automatic weather station (Met One Instruments, Inc., USA) at Yueyang.

**Figure 2:**
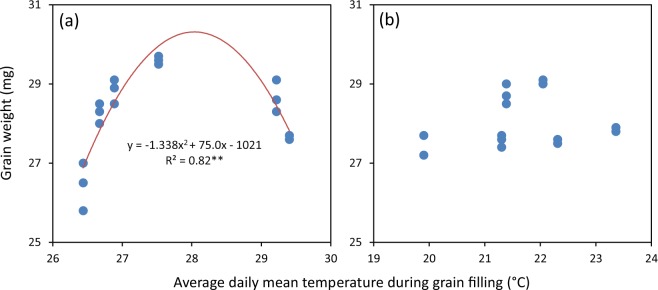
Relationship between grain weight and average daily mean temperature during grain filling in the early- (**a**) and late-season rice (**b**). The data used for analysis are presented in Table 2 and Fig. 1. Data analysis was performed by linear regression analysis (Statistix 8, Analytical Software, Tallahassee, FL, USA). ^**^ denotes significant relationship at the 0.01 probability level.

There were considerable variations in grain yield and yield attributes across seasons, sites and cropping cycles (or years), even for the relatively stable yield component – grain weight. These variations were partly attributable to variations in climatic conditions such temperature (Fig. 1a–d). For example, grain weight varied largely (from 25.8 to 29.7 mg) in the early-season rice with high temperature during grain filling and there was a significant quadratic relationship between grain weight and average daily mean temperature during grain filling (Fig. 2a). In the late-season rice with relatively low temperature during grain filling, the variation in grain weight was relatively small (from 27.2 to 29.1 mg) and no significant relationship was observed between grain weight and average daily mean temperature during grain filling (Fig. 2b). These observations also indicate that grain weight may be more sensitive to high temperature than low temperature in rice.

## Conclusions

Growing oilseed rape in the fallow season could improve both sink size and source capacity and consequently increase grain yield in machine-transplanted double-season rice. The improved sink size was mainly attributed to increased panicle size and panicle number for early- and late-season rice, respectively. Further investigations are required to examine the effect on yield components using cultivars with different tillering capacities and panicle sizes and determine the soil and physiological processes for the improved source capacity.

## Methods

### Sites and soils

Field experiments were conducted at Hengyang (26°53′ N, 112°28′ E) and Yueyang (29°16′ N, 113°05′ E), Hunan Province, China from 2014 to 2016. The two sites have a moist subtropical monsoon climate. Double-season rice (i.e. early- and late-season rice) cropping is a major rice-based system at both sites. Leaving the land fallow or growing Chinese milk vetch is usually made during the following non-rice season. Daily temperatures tend to increase during the early rice-growing season, whereas they tend to decrease during the late rice growing season (Fig. 1a–d).

The soil of the Hengyang site was an Ultisol (USDA taxonomy) with the following properties: pH = 6.24, organic matter = 18.4 g kg^−1^, total N = 2.66 g kg^−1^, total P = 0.73 g kg^−1^, and total K = 6.55 g kg^−1^. The soil of the Yueyang site was a Fluvent with the following properties: pH = 6.52, organic matter = 19.6 g kg^−1^, total N = 2.61 g kg^−1^, total P = 0.76 g kg^−1^, and total K = 7.16 g kg^−1^. Soil tests were based on samples taken from the upper 20 cm of the soil before the experiment started in 2014. The pH was determined using a digital pH meter (Model 868, Thermo Orion, MA, USA), organic matter was measured using the potassium dichromate method, total N was obtained with the semi-micro Kjeldahl method, total P was determined with the molybdenum blue method, and total K was measured using a flame photometer (FP640, Shanghai Precision & Scientific Instrument Inc., Shanghai, China).

### Experimental design and crop management

Three cropping systems were established after harvesting the late-season rice in 2014 at each site: (1) oilseed rape followed by double-season rice (ODR), (2) Chinese milk vetch followed by double-season rice (MDR), and (3) fallow followed by double-season rice (FDR). The cropping systems were arranged in a randomized complete-block design with four replications. The plot size was 45 m^2^ in Hengyang and 35 m^2^ in Yueyang. The plot arrangement was maintained for the duration of the experiment.

A short-duration (about 180 d) oilseed rape cultivar Xiangyou 104 was used in the experiment. The oilseed rape was planted and managed according to recommendations of the seed supplier (Oil Crop Research Institute, Hunan Agricultural University). Namely, the oilseed rape was grown under no-tillage direct seeding (manual broadcasting) after harvesting the late-season rice at a seeding rate of 9 kg ha^−1^. Oilseed rape plants received 120 kg N ha^−1^, 67.5 kg P_2_O_5_ ha^−1^, and 120 kg K_2_O ha^−1^ in each season. The N fertilizer was applied in three splits: 50% as basal fertilizer, 20% at the 5 leaf stage, and 30% at flower bud emergence. The P fertilizer was applied as basal fertilizer. The K fertilizer was split equally as basal fertilizer and at flower bud emergence. Pathogens, insects, and weeds were controlled by chemicals to avoid yield loss. Chinese milk vetch was grown under no-tillage direct seeding at a seeding rate of 30 kg ha^−1^, without application of chemical fertilizer. The oilseed rape straw and the whole plant of Chinese milk vetch were incorporated into the soil during land preparation (plowing and harrowing) for the early-season rice cultivation. No management activities were employed for fallow plots. All plots were plowed at about 5 days before transplanting the early-season rice.

An inbred rice cultivar, Zhongzao 39, was grown in both the early and late seasons. Zhongzao 39 is an *indica* rice cultivar developed by the China National Rice Research Institute with Jiayu 253 as the female parent and Zhongzu 3 as the male parent. This cultivar has moderate tillering capacity and medium plant height. Zhongzao 39 has been widely grown by rice farmers in the study regions. Pre-germinated seeds were sown in trays (length × width × height = 58 cm × 25 cm × 2 cm) at a rate of 130 g tray^−1^ on 12 April in the early season and on 10 July in the late season. Seedlings of about 25- and 15-days-old were transplanted in the early and late seasons, respectively. The different seedling ages used in the two seasons was due to the difference in temperature during the seedling growing period, which was lower in the early season than in the late season. Transplanting was done at a hill spacing of 25 cm × 11 cm with 7–8 seedlings per hill, using a high-speed rice transplanter (PZ80-25, Dongfeng Iseki Agricultural Machinery Co., Ltd., Xiangyang, China). All plots received 120 kg N ha^−1^, 67.5 kg P_2_O_5_ ha^−1^, and 120 kg K_2_O ha^−1^ in each season. The N fertilizer was applied in three splits: 50% as basal fertilizer, 20% at early tillering (7 days after transplanting), and 30% at panicle initiation. The P fertilizer was applied as basal fertilizer. The K fertilizer was split equally as basal fertilizer and at panicle initiation. The strategy for water management was in the sequence of flooding, midseason drainage, re-flooding, moist intermittent irrigation and drainage. Pathogens, insects, and weeds were controlled by chemicals to avoid yield loss. The rice straw was returned to the plot.

### Sampling and measurements

Ten hills of rice plants were sampled diagonally from a 5-m^2^ harvest area for each plot at maturity. Panicle number per hill was counted to calculate panicle number per m^2^. The plant samples were separated into straw (including rachis) and grains by hand threshing. Filled spikelets were separated from unfilled spikelets by submergence in tap water. Three subsamples of 30 g filled spikelets and all unfilled spikelets were counted to calculate spikelet number per panicle, spikelet number per m^2^, and spikelet filling percentage. Dry weights of straw and of filled and unfilled spikelets were determined after oven-drying at 70 °C to a constant weight. Grain weight, total biomass, and harvest index were calculated. Grain yield was determined from a 5-m^2^ area in each plot and adjusted to a moisture content of 14%.

### Statistical analysis

All data were analyzed using analysis of variance (Statistix 8, Analytical Software, Tallahassee, FL, USA). The statistical model for the analysis of variance included replication, cropping system, site, cropping cycle, the two-factor interactions of cropping system × site, cropping system × cropping cycle and cropping cycle × site, and the three-factor interaction of cropping system × site × cropping cycle. The least significant difference (LSD) test was used following the analysis of variance to evaluate the significance of differences among means of cropping systems. Statistical significance was set at the 0.05 probability level.

## Data Availability

All data generated or analysed during this study are included in the article.

## References

[1] Muthayya S, Sugimoto JD, Montgomery S, Maberly GF (2014). An overview of global rice production, supply, trade, and consumption. Ann. N. Y. Acad. Sci..

[2] Buresh, R. J., Larazo, W. M., Laureles, E. V., Samson, M. I. & Pampolino, M. F. Sustainable soil management in lowland rice ecosystems in *Organic-based agriculture for sustained soil health and productivity* (eds Javier, E. F., Mendoza, D. M. & dela Cruz N. E.) 116–125 (Central Luzon State University, 2005).

[3] Ray DK, Foley JA (2013). Increasing global crop harvest frequency: recent trends and future directions. Environ. Res. Lett..

[4] Joshi AK, Chand R, Arun B, Singh RP, Ortiz R (2007). Breeding crops for reduced-tillage management in the intensive, rice-wheat systems of South Asia. Euphytica.

[5] Ladha JK (2003). How extensive are yield declines in long-term rice-wheat experiments in. Asia? Field Crops Res..

[6] Zegada-Lizarazu W, Monti A (2011). Energy crops in rotation. A review. Biomass Bioenerg..

[7] International Rice Research Institute. World rice statistics database, http://ricestat.irri.org:8080/wrsv3 (2018).

[8] Zou Y (2011). Development of cultivation technology for double cropping rice along the Changjiang river valley. Sci. Agr. Sin..

[9] Peng S (2014). Reflection on China’s rice production strategies during the transition period. Sci. Sin. Vitae.

[10] Huang M, Zou Y (2018). Integrating mechanization with agronomy and breeding to ensure food security in China. Field Crops Res..

[11] Yang Z, Zheng S, Nie J, Liao Y, Xie J (2014). Effects of long-term winter planted green manure on distribution and storage of organic carbon and nitrogen in water-stable aggregates of reddish paddy soil under a double-rice cropping system. J. Integr. Agr..

[12] Lee CH (2010). Effect of Chinese milk vetch (*Astragalus sinicus* L.) as a green manure on rice productivity and methane emission in paddy soil. Agr. Ecosyst. Environ..

[13] Xie Z (2017). Chinese milk vetch improves plant growth, development and ^15^N recovery in the rice-based rotation system of south China. Sci. Rep..

[14] Zhou C (2016). Integration of growing milk vetch in winter and reducing nitrogen fertilizer application can improve rice yield in double-rice cropping system. Rice Sci..

[15] Cao W (2017). Reviews and prospects on science and technology of green manure in China. J. Plant Nutr. Fertil..

[16] Huang M, Shan S, Cao F, Chen J, Zou Y (2018). The potential of naturally occurring fallow weeds to scavenge nitrogen in rice cropping systems. Ecol. Indic..

[17] Kirkegaard JA, Hocking PJ, Angus JF, Howe GN, Gardner PA (1997). Comparison of canola, Indian mustard and Linola in two contrasting environments. II. Break-crop and nitrogen effects on subsequent wheat crops. Field Crops Res..

[18] Huang M (2016). Rice yield and the fate of fertilizer nitrogen as affected by addition of earthworm casts collected from oilseed rape fields: A pot experiment. Plos One.

[19] Huang, M. *et al*. Increased soil fertility in a long-term rice-oilseed rape cropping system and its potential roles in reducing nitrogen inputs and environmental impacts in Cropping systems: applications, management and impact (ed. Hodges, J. G.) 103–113 (Nova Science Publishers, 2017).

[20] Huang M (2018). Earthworm responses to cropping rotation with oilseed rape in no-tillage rice fields and the effects of earthworm casts on human-essential amino acid content in rice grains. Appl. Soil Ecol..

[21] Guan C, Chen S, Wu M (2010). Research evolution on breeding and mechanical cultivation of early-mature winter rapeseed in double-crop rice area in southern China. Chin. J. Eng. Sci..

[22] Huang M (2013). Yield gap analysis of super hybrid rice between two subtropical environments. Aust. J. Crop Sci..

[23] Ying J (1998). Comparison of high-yield rice in tropical and subtropical environments I. Determinants of grain and dry matter yields. Field Crop Res..

[24] Zhang Y (2009). Yield potential and radiation use efficiency of “super” hybrid rice grown under subtropical conditions. Field Crops Res..

[25] Huang M (2011). No-tillage and direct seeding for super hybrid rice production in rice-oilseed rape cropping system. Eur. J. Agron..

[26] Yang J, Zhang J (2006). Grain filling of cereals under soil drying. New Phytol..

